# RAMP2-AS1 Regulates Endothelial Homeostasis and Aging

**DOI:** 10.3389/fcell.2021.635307

**Published:** 2021-02-12

**Authors:** Chih-Hung Lai, Aleysha T. Chen, Andrew B. Burns, Kiran Sriram, Yingjun Luo, Xiaofang Tang, Sergio Branciamore, Denis O’Meally, Szu-Ling Chang, Po-Hsun Huang, John Y-J. Shyy, Shu Chien, Russell C. Rockne, Zhen Bouman Chen

**Affiliations:** ^1^Department of Diabetes Complications and Metabolism, City of Hope, Duarte, CA, United States; ^2^Cardiovascular Center, Taichung Veterans General Hospital, Taichung, Taiwan; ^3^Institute of Clinical Medicine, National Yang-Ming University, Taipei, Taiwan; ^4^Irell and Manella Graduate School of Biological Sciences, City of Hope, Duarte, CA, United States; ^5^Center for Gene Therapy, City of Hope, Duarte, CA, United States; ^6^Department of Medicine, University of California, San Diego, La Jolla, CA, United States; ^7^Department of Bioengineering, University of California, San Diego, La Jolla, CA, United States; ^8^Division of Mathematical Oncology, Department of Computational and Quantitative Medicine, City of Hope, Duarte, CA, United States

**Keywords:** endothelial function, aging, RAMP2-AS1, RAMP2, lncRNA, transcriptome, PCA, shear stress

## Abstract

The homeostasis of vascular endothelium is crucial for cardiovascular health and endothelial cell (EC) aging and dysfunction could negatively impact vascular function. Leveraging transcriptome profiles from ECs subjected to various stimuli, including time-series data obtained from ECs under physiological pulsatile flow vs. pathophysiological oscillatory flow, we performed principal component analysis (PCA) to identify key genes contributing to divergent transcriptional states of ECs. Through bioinformatics analysis, we identified that a long non-coding RNA (lncRNA) RAMP2-AS1 encoded on the antisense of RAMP2, a determinant of endothelial homeostasis and vascular integrity, is a novel regulator essential for EC homeostasis and function. Knockdown of RAMP2-AS1 suppressed RAMP2 expression and caused EC functional changes promoting aging, including impaired angiogenesis and increased senescence. Our study demonstrates an integrative approach to quantifying EC aging based on transcriptome changes, which also identified a number of novel regulators, including protein-coding genes and many lncRNAs involved EC functional modulation, exemplified by RAMP2-AS1.

## Introduction

Vascular endothelium constitutes the vital interface between circulating blood and the vascular wall. The homeostasis of vascular endothelial cells (ECs), including their vasodilatory function and anti-inflammatory, anti-coagulatory, and anti-thrombotic properties, is crucial to vascular health (Fisher et al., 2001). In the process of aging, ECs undergo functional declines, which is manifested by a constellation of cellular and molecular changes, e.g., attenuated endothelium-dependent vasodilation, increased reactive oxygen species (ROS) production, and impaired angiogenic and regenerative capacities. This process of EC aging has been reviewed and discussed and various genes and pathways have been proposed to be important players (Brandes et al., 2005; Donato et al., 2015; Ungvari et al., 2018). However, the rate and the extent of EC aging have not been experimentally investigated at the transcriptome level using quantitative measurements.

In order to address this question, one approach would be to use a longitudinal time-series transcriptome data collected from ECs undergoing an aging-related process. One such process is EC response to flow, evident by the focal distribution of atherosclerotic lesions that are aggravated by aging (Wang and Bennett, 2012; Gimbrone and Garcia-Cardena, 2016). For example, the laminar and pulsatile shear stress (PS) in the straight parts of the artery not only causes an elevated expression of anti-oxidative and anti-inflammatory genes, but also suppresses pro-oxidative and pro-inflammatory genes, thus contributing to a potential anti-aging effect. Indeed, several key regulators in aging, e.g., Sirtuins 1 and 3 and genes supporting the mitochondrial antioxidant defense systems, e.g., PGC1α and Nrf1, are upregulated by laminar flow or PS (Chen et al., 2010; Tseng et al., 2014; Wu et al., 2018). In contrast, the oscillatory shear stress (OS) due to disturbed flow in the curvatures and bifurcations strongly induces these pro-oxidative and pro-inflammatory genes while suppressing the antioxidative and anti-inflammatory genes that cause EC dysfunction and predispose vascular disease (see Malek et al., 1999; Harrison et al., 2003; Chiu and Chien, 2011 for review).

Using a parallel-plate flow system, we have previously profiled the transcriptomes using RNA-sequencing (RNA-seq) in cultured human ECs subject to PS and OS in a time-course encompassing transient and chronic transcriptional changes (Ajami et al., 2017; Miao et al., 2018; He et al., 2019). Consistent with the well documented divergent effects, compared to OS, PS induced an anti-inflammatory, anti-oxidative, and anti-fibrotic gene expression program. In addition, leveraging these RNA-seq data, we have also identified several novel EC regulators, including several long non-coding RNAs (lncRNAs) as essential epigenetic components of EC response to flow (Huang et al., 2017; Miao et al., 2018).

In this study, we propose that PS deters EC aging by regulation of a transcriptional program underlying an anti-EC aging effect. We first leveraged temporal measurements of transcriptome (i.e., shear stress time-course RNA-seq) data to model a compressed aging trajectory associated with PS or OS through principal component analysis (PCA). Next, through exploring the key genes engaged in the distinct effects of PS vs. OS, as well as other stimuli known to regulate EC function, i.e., pro-inflammatory cytokine tumor necrosis factor alpha (TNFα) and endothelial protective drug statins, we identified receptor activity-modifying protein 2 (*RAMP2*, a known key regulator in EC function) and *RAMP2-AS1* (a lncRNA transcribed from the anti-sense of RAMP2) as a putative pair of genes that contribute to the divergence in EC aging trajectories. We further investigated the functional importance of RAMP2-AS1 in the regulation of RAMP2 and EC function. Together, our study demonstrates an integrative approach to unraveling novel crucial regulators and regulatory mechanisms in EC aging.

## Materials and Methods

### Aging Trajectory and Eigengene Analysis

We have previously published the PS and OS EC RNA-sequencing (RNA-seq) data (GSE103672) (Ajami et al., 2017; Miao et al., 2018), consisting of 10 timepoints (i.e., 0, 1, 2, 3, 4, 6, 9, 12, 16, and 24 h), each with two biological replicates for both the PS and OS conditions. We constructed a data matrix (*X*) of log2 transformed counts per million reads (CPM) and performed PCA on the mean-centered data matrix with the singular value decomposition method so that *X* = *U*Σ*V*^∗^. Matrices were created for protein-coding genes (PCGs) and non-coding genes (NCGs) and were analyzed separately. Through an analysis of all principal components, we identified components which resulted in the most separation, and hence explained most of the variance, between the PS and OS conditions. These components were identified as PC3 for PCGs and PC2 for NCGs. We then constructed aging trajectories by plotting these components over time as performed in our previous study (Rockne et al., 2020; Supplementary Figure 1).

Genes associated with aging were defined by the principal component loadings (right singular vectors in the matrix *V*^∗^) corresponding to the aging trajectories, referred to as eigengenes. The eigengenes were ranked in descending order by absolute value (Supplementary Table 1) and were used to project additional RNA-seq data (X’) into the component space by the transformation X’V. Coding and non-coding eigengenes were also ranked by mutual information with respect to the flow condition using a method for combining discrete and continuous data (Gao et al., 2017). Mutual information measures the mutual dependence between variables and quantifies the amount of information obtained about one variable by observing the other, in this case between genes and the flow conditions. The top 50-ranked protein-coding and non-coding eigengenes are listed in Supplementary Table 3.

### EC Culture, Treatment, and Transfection

HUVECs (Cell Applications Inc., San Diego, CA) (Passages 4–8) from pooled donors were cultured in HUVEC growth medium containing EC growth supplements (Cell Applications Inc.), based on the manufacturer’s recommendations, at 37°C in an atmosphere of 95% air and 5% CO_2_. HUVECs were treated with atorvastatin (ATV) at 1 or 5 mM, or TNFα at 100 ng/ml for 24 h. Antisense LNA GapmeRs specifically targeting two different regions of RAMP2-AS1 (LG00116686, LG00226723) and scramble control designed and purchased from Qiagen were transfected into ECs with Lipofectamine 3000 following the protocol provided by the manufacturer and as described previously (Miao et al., 2018).

### Intima Samples From Human Mesenteric Arteries

Intimal RNA was isolated from deidentified human mesenteric arteries obtained from the organ donors of Southern California Islet Cell Resource Center at City of Hope. The research consents for the use of postmortem human tissues were obtained from the donors’ next of kin and ethical approval for this study was granted by the Institutional Review Board of City of Hope (IRB #01046). Intimal RNA was collected by flushing once the inner lumen with TRIzol following an established method (Nam et al., 2009) and as previously described (Tang et al., 2020).

### RNA Extraction, Quantitative PCR, Library Preparation

RNA was extracted from cells and tissues using TRIzol (Invitrogen) following the manufacturer’s instructions as described previously (Miao et al., 2018; Tang et al., 2020). The total RNA was reverse transcribed using the PrimeScript^TM^ RT Master Mix (Cat# RR036A-1, Takara), and cDNAs were used for PCR and qPCR analyses using Biorad CFX96. Each qPCR sample was performed in triplicate, with iTaq Universal SYBR Green Supermix (BioRad). β-actin (ACTB) was used as the internal control. For RNA-seq, 500 ng of total RNA per sample was used and the libraries were prepared using the KAPA mRNA HyperPrep Kit (Roche Diagnostics) following the manufacturer’s manual. The libraries were sequenced with HiSeq2500 using the SR50 mode.

### Immunoblotting

Upon collection, total cell protein was extracted with a NP40 Cell Lysis Buffer (Thermo Fisher Scientific) in the presence of a protease inhibitor (Cat#8340; Sigma-Aldrich) and a phosphatase inhibitor cocktail (Cat# 5870; Cell Signaling). Protein extracts (30 μg), along with a protein ladder, were loaded onto a 15% SDS-PAGE gel followed by transferring onto a PVDF membrane. The membranes were blocked with 5% skim milk and then incubated with primary antibodies in 3% BSA overnight at 4°C. The primary antibodies used were mouse anti-RAMP2 (sc-365240, Santa Cruz 1:100 dilution) and rabbit anti-β-actin (8457S, Cell Signaling, 1:1,000 dilution), which was used as a loading control. After washing with TBST (containing 0.1% Tween-20), the membranes were then incubated with HRP-conjugated anti-rabbit (7074S, Cell Signaling) or anti-mouse (7076S, Cell Signaling) secondary antibodies at room temperature for 1 h and developed using ECL substrate (Cat# WBKLS0500, Millipore). The densitometry was analyzed with use of Image J.

### RNA-Seq Data Analysis

STAR (Dobin et al., 2013) was used to align raw sequencing data to the GRCh38 reference using GENCODE annotation Release 33. Each library was subjected to extensive quality control, including estimation of library complexity, gene body coverage, and duplication rates, among other metrics detailed in the pipeline repository. Reads were counted across genomic features using Subread featureCounts (Liao et al., 2013) and merged into a matrix of counts per gene for each sample. DESeq2 (Love et al., 2014) was then used to perform differential expression analysis with default parameters. Gene ontology enrichment analysis was performed through the Gene Ontology Consortium platform (Ashburner et al., 2000) and Benjamini-Hochberg corrected *P* < 0.05 were considered significantly enriched pathways.

### Senescence-Associated β-gal (SA β-gal) Staining

Cytochemical staining for SA-β-galactosidase was performed using the Senescence β-Galactosidase Staining Kit (Cell Signaling Technology) following the manufacturer’s manual. Briefly, the ECs following transfection were washed once with freshly prepared 1 × PBS, fixed in 1X Fixative solution for 10–15 min at room temperature, and then rinsed twice with 1 × PBS. The cells were stained for 48 h in a dry incubator before viewing under an Olympus JP/1 × 71 fluorescence microscope (Olympus, Japan) with digital camera output. The percentage of SA-β-galactosidase positive cells was determined by counting the number of blue cells under bright field illumination. The positively stained cells in three randomly selected low-power fields per well were counted by an independent observer in a blind fashion. The average was taken from the three fields.

### Apoptosis Analysis

EC apoptosis was assayed using the FITC Annexin V Apoptosis Detection Kit II (BD Pharmingen, Cat # 556570) following the manufacturer-recommended protocol. Briefly, cells were collected using 0.05% Trypsin-EDTA, washed once with PBS and resuspended in the binding buffer. Cells were then stained with 5 μL Propodium Iodide Staining solution and 5 μL FITC Annexin V and incubated in the dark at room temperature of 20 min. Following incubation, cells were immediately analyzed on Accuri C6 flow cytometry (BD Biosciences).

### Hanging Drop Cell Aggregation and 3D Sprouting Assay

Three-dimensional (3D) spheroid sprouting assay was performed following a published protocol (Heiss et al., 2015) with modifications. ECs cultured to confluency were trypsinized for 5 min at 37°C using 0.25% Trypsin/0.53 mM EDTA (ATCC 30-2101) and counted. The cell suspension was neutralized with M199 media and centrifuged at 200 rcf for 5 min. The media was aspirated, and the cells were resuspended at 10^6^ cells/mL in fresh media. To form homogeneous aggregates of 500 cell per aggregate, 125 μl of the resuspended cells were added to 3.875 mL of M199 media. One mL of 0.3% (W/V) methylcellulose (Sigma M0512-100G) in M199 media was then added to the suspension to bring the total volume to 5 mL, resulting in a final density of 500 cells per 20 μL. The cell suspension was then distributed onto the inside lid of a petri dish using a multichannel pipette to form rows of 20 μL droplets. The dish was then inverted, and 5 mL of PBS was added to the bottom of the dish. Cells were incubated overnight. On the next day, cell aggregates were collected, washed, and centrifuged at 100 rcf for 2.5 min. The aggregates were resuspended in pre-chilled Matrigel (Corning 356234) to allow for two aggregates per 20 μL. Aggregates were distributed in 20 μL droplets onto the bottom of the plate. The plate was then turned upside down to form hanging drops and placed into a larger petri dish (to maintain sterility), which was then placed in incubator for 30 min. After the Matrigel has begun to gel, the plate was removed from the larger dish and turned upright to allow another hour to fully set. The gelled aggregates were then overlaid with M199 media containing 50 ng/mL of VEGF (Sigma V7259-10UG) and incubated for 3 days and monitored for sprouting.

Brightfield images of the sprouts were taken using an Amscope MU1000 camera and an Olympus IX50 microscope at 10× magnification. The images were then analyzed in FIJI (ImageJ) using the Sprout Morphology analysis tool (Eglinger et al., 2017). The images were first converted to 8-bit binary masks. Threshold values were then globally adjusted across all images to uniformly darken the background and highlight the aggregates and their sprouts. The images were then manually assessed for bubbles or other artifacts in the gel that the software could misconstrue as an aggregate. Pixel scale was determined using a hemocytometer, and this was applied globally to all images. Then the images were batch-run through the analysis package, where the software measured the aggregate and the sprout network. Data was exported from FIJI for statistical analysis.

### Statistical Analysis

Statistical analysis was performed using Student’s *t*-test (two-sided) between two groups or ANOVA followed by Tukey’s post-test for multiple-group comparisons. If variances between two groups were significantly different (F-test), non-parametric Mann–Whitney *U*-test was applied. *P* < 0.05 was considered as statistically significant. For all the experiments, at least three independent experiments were performed unless otherwise specified.

## Results

### ECs Under PS and OS Show Divergent Aging Trajectories Through Transcriptome Analysis

**FIGURE 1:**
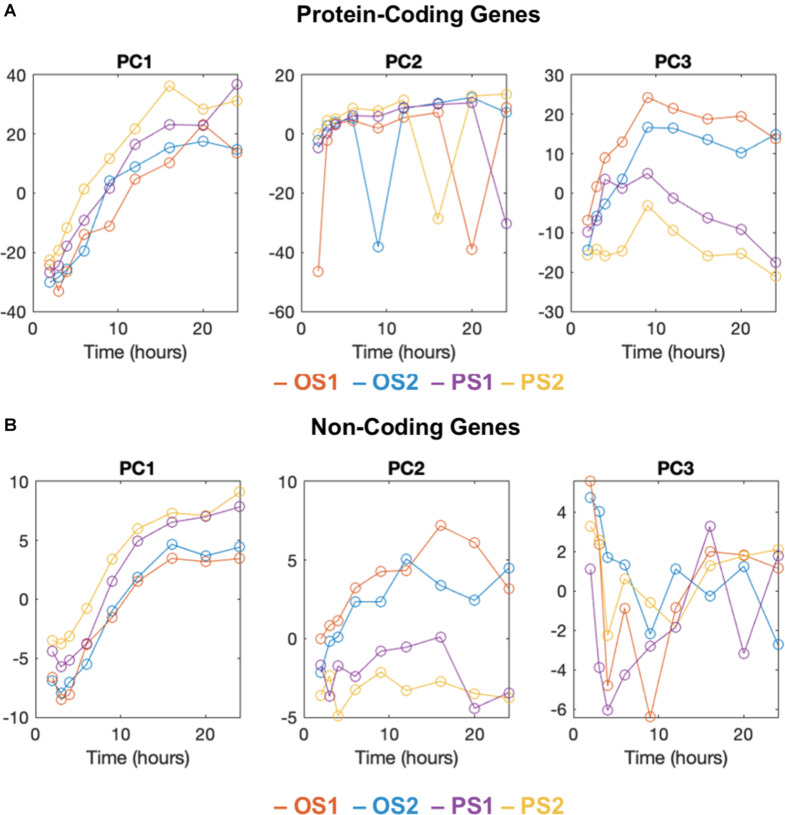
PCA analysis and aging trajectory of ECs subject to PS and OS. The first 3 principal components of time-series RNA-sequencing data plotted against time (hours) for protein coding genes **(A)** and non-coding genes **(B)** of two replicates (OS 1, 2; PS 1, 2). **(A)** The first principal component (PC1) is correlated with time. The second component (PC2) includes intermittent spiking signals associated with the expression of mitochondrial genes, and the third component (PC3) shows divergent trajectories between OS and PS conditions. **(B)** Same as A except for non-coding genes. Divergent trajectories are shown in the second component (PC2). The third component is not clearly interpretable.

To construct an aging trajectory in ECs responding to PS and OS, we leveraged the RNA-seq data previously obtained from ECs subject to the PS (12 ± dyne/cm^2^) and OS (1 ± 5 dyne/cm^2^) for 10 time points (i.e., 0, 1, 2, 3, 4, 6, 9, 12, 16, and 24 h) (Ajami et al., 2017; Miao et al., 2018). For PCGs, PCA identified that the majority of the variance in these data was contained in the first 8 PCs (Supplementary Figure 1A). When plotting the first 8 PCs *vs.* time, we found that PC1 correlated with time, PC2 exhibited intermittent spiking signals associated with the expression of mitochondrial genes, and PC3 showed divergent trajectories between OS and PS (Figure 1A and Supplementary Figures 1B,C). In contrast, PC4 to PC8 were not clearly interpretable (Supplementary Figure 1C). Similarly, we performed PCA for the NCGs, particularly long intergenic non-coding RNAs (lincRNAs), which revealed divergent trajectories of PS *vs.* OS over time in PC2. To this end, we identified PC3 in PCGs and PC2 in NCGs to be reflective of divergent aging trajectories between PS and OS conditions.

To evaluate the potential of using PCA of PCGs in assessing the aging state of ECs, we used the PC3 eigengenes to project RNA-seq data obtained from HUVECs-treated with atorvastatin (ATV) that has been shown to exert EC protective effects (Sun et al., 2006; Chen et al., 2009; He et al., 2017) and those stimulated by TNFα that causes EC dysfunction (Zhang et al., 2009) (GSE163433). The PCA revealed that atorvastatin (ATV)-treated cells were similar to PS-imposed cells, whereas those treated with TNFα were more like those under OS (Supplementary Figure 2A). We also used RNA-seq data from hypoxia-treated human microvascular ECs we have published (GSE136912) (Tang et al., 2020), which appeared to be in-between of PS and OS regardless of the duration (Supplementary Figure 2B). This result is in line with a lack of clearly expected effect of hypoxia in EC aging.

### LncRNA RAMP2-AS1 Is a Candidate Gene Contributing to Divergent Aging Trajectories

**FIGURE 2:**
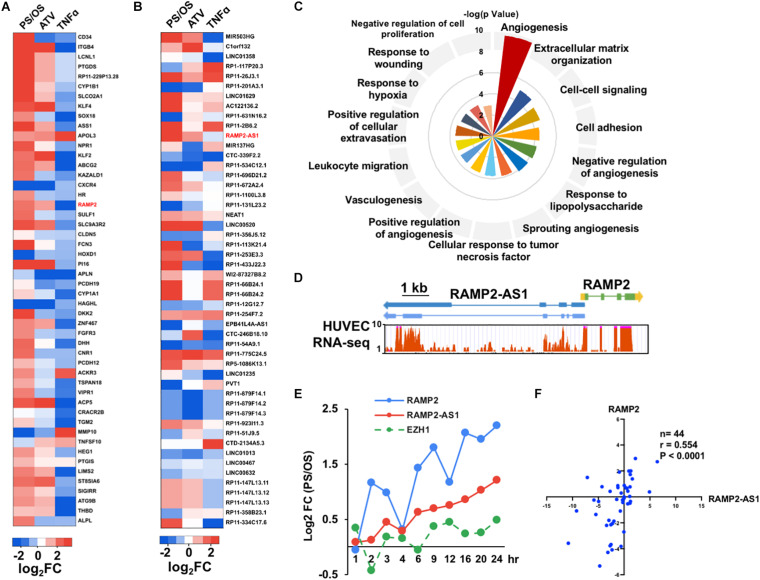
Identification of RAMP2 and RAMP2-AS1 as top candidates. **(A,B)** PS/OS, ATV/control, and TNFα/control log2 fold changes in expression of top 50 protein-coding genes (PCGs), ranked by absolute values of PC3; non-coding genes (NCGs), ranked by absolute values of PC2. RAMP2 and RAMP2-AS1 are labeled in red showing induction by PS and ATV and suppression by TNFα. **(C)** Top 15 Biological Pathway terms from pathway enrichment analysis of top 200 PCGs, plotted with -log_10_ *P*-value. **(D)** Genomic structures of RAMP2-AS1 and RAMP2, wherein blocks depict exons, horizontal lines resemble introns, and arrowheads indicate the opposite transcriptional directions of RAMP2-AS1 and RAMP2; aligned with RNA-seq measurements along RAMP2 and RAMP2-AS1 in HUVECs. **(E)** RNA levels of RAMP2, RAMP2-AS1, and EZH1, plotted with PS/OS log2 fold changes across 10 time points, from 1 to 24 h under PS or OS. **(F)** Spearman correlation between RAMP2-AS2 and RAMP2 RNA levels in 44 human mesenteric arterial intimae quantified by qPCR, plotted with log2-transformed relative gene expression.

To evaluate the relative contribution of various genes to the aging trajectory and identify novel EC regulators, we ranked both PCGs and NCGs using PCA (Supplementary Tables 1, 2). Among the top ranked PCGs, several are well characterized to be crucial for EC responses to PS, e.g., *KLF4* (ranked #8), *ASS1* (ranked #10), *KLF2* (ranked #13), *THBD* (ranked #49), and *eNOS* (ranked #63) (Dekker et al., 2002; Lin et al., 2005; Parmar et al., 2006; Hamik et al., 2007; Fang and Davies, 2012; Figure 2A). Pathway enrichment analysis of the top 200 ranked PCGs from PC3 indicated that they are involved in key functions of ECs, including angiogenesis, extracellular matrix organization, and cell adhesion (Figure 2C). Among the top-ranked NCGs (Figure 2B), MIR503HG (host gene of miR-503, ranked #1) has been shown to be important for cell cycle control and angiogenic function in ECs (Fiedler et al., 2015) and Clorf132 (ranked #2) is the host gene of miR-29b and miR-29c of which DNA methylation has been identified as a marker for prediction of chronological age (Zbieæ-Piekarska et al., 2015; Cho et al., 2017). Noticeably, LINC00520 (aka LEENE), which we previously identified as a PS-induced lncRNA that enhances eNOS expression (Miao et al., 2018), ranked #20. These data suggest that the PCA indeed identified genes known to be important for EC function or have been implicated in aging.

Next, we focused on identifying novel lncRNA regulators in EC biology because there is much less known about lncRNA functions. To further prioritize the lncRNAs candidates, we cross-referenced the shear stress data with those derived from ECs-treated with ATV or TNFα. Specifically, we screened for the top NCGs from the PCA of shear stress datasets with the following criteria: fold change (FC) showing (1) positive association between PS/OS and ATV/control and (2) negative association between PS/OS and TNFα/control. The top three lncRNAs were the two miRNA host genes MIR503HG and C1orf132, and RAMP2-AS1 (Supplementary Figure 3).

### Co-expression Pattern of RAMP2-AS1 and RAMP2 in ECs

The *RAMP2-AS1* gene is located on the antisense strand of the *RAMP2* gene (chr 17:40, 894, 172-40, 919, 137), which encodes receptor activity-modifying protein 2 (RAMP2), an adrenomedullin (ADM)-receptor accessory protein. *RAMP2-AS1* is transcribed divergently from *RAMP2*, with its transcription start site immediately upstream to that of *RAMP2* (Figure 2D). Interestingly, following the initial PCA, both RAMP2 and RAMP2-AS1 are highly ranked (#19 for RAMP2 in the PCG list and #11 for RAMP2-AS1 in the NCG list). Furthermore, RAMP2 has been demonstrated to be essential for EC function and vascular integrity as EC deletion of RAMP2 led to impaired angiogenic capacity as well as accelerated vascular senescence (Ichikawa-Shindo et al., 2008; Koyama et al., 2013).

Examining data available in Genotype-Tissue Expression (GTEX) project, we found similar tissue-specific gene expression patterns of RAMP2 and RAMP2-AS1 (Supplementary Figure 4). Both RAMP2 and RAMP2-AS1 mRNA levels were increased by PS in a time-dependent manner, when compared with OS (Figure 2E). Furthermore, based on multiple EC RNA-seq datasets (including shear stress, ATV, and TNFα), the mRNA levels of *RAMP2* and *RAMP2-AS1* showed a strong positive correlation (*R* = 0.87, *P* < 0.001), which was not observed for *RAMP2-AS1* and *EZH1* encoded less than 9 kb downstream of RAMP2-AS1 (*R* = 0.36, *P* = 0.302) (Figure 2E). We also used qPCR to confirm the co-regulatory pattern of RAMP2 and RAMP2-AS1 in HUVECs. For initial trial, we designed 4 sets of primers flanking 4 different exons of RAMP2-AS1 transcripts, which produced consistent data (Supplementary Figures 5A,B). For the rest of the experiments, we used the primer set that specifically amplifies a fragment in Exon 4 common to both RAMP2-AS1 mature transcripts. Consistent with the RNA-seq data, qPCR confirmed that ATV increased, whereas TNFα substantially decreased RAMP2 and RAMP2-AS1 RNA levels in ECs (Supplementary Figures 5B–D). Importantly, in mesenteric arterial endothelium isolated from 44 human donors (see donor characteristics in Supplementary Figure 6), RAMP2-AS1 and RAMP2 were also positively correlated (*r* = 0.55, *P* < 0.001, Figure 2F). Together, these data underscored a potential regulatory link between RAMP2 and RAMP2-AS1.

### RAMP2-AS1 Regulates RAMP2 in ECs

**FIGURE 3:**
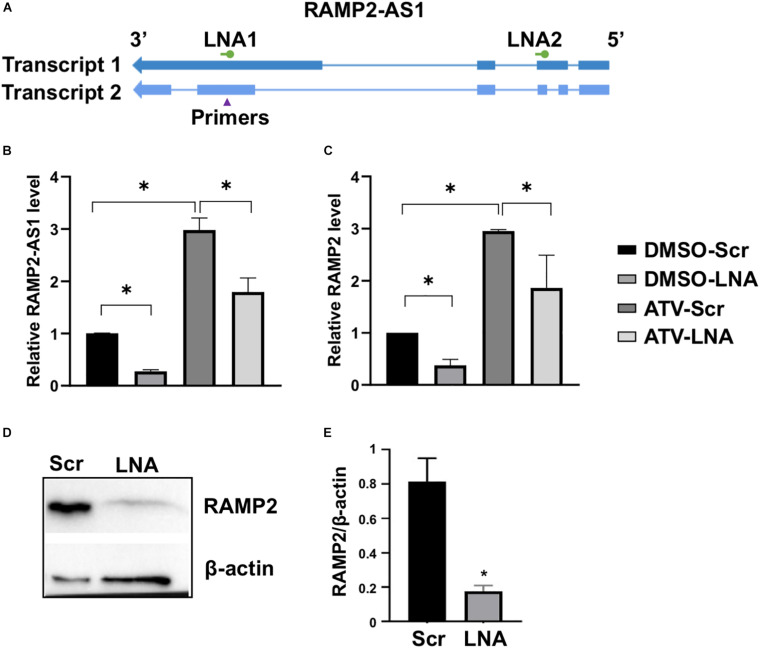
RAMP2-AS1 inhibition suppresses RAMP2 expression. **(A)** Design of LNAs and qPCR primers for RAMP2-AS1 transcripts. **(B,C)** HUVECs were transfected with scramble (Scr) or locked nucleic acid (LNA) GapmeRs against RAMP2-AS1 (LNA1, hereafter labeled “LNA”) at 25 nM, before treatment with ATV (5 μM) or DMSO. RAMP2-AS1 and RAMP2 mRNA levels were analyzed by qPCR. **(D,E)** Baseline HUVEC transfected with Scr and LNA were subjected to immunoblotting with specific antibodies as indicated. Representative blots **(D)** and densitometry analysis based on three experiments **(E)**. Data are representative of mean ± SEM from 5 **(B,C)** and 3 **(D,E)** independent experiments with each experiment performed in duplicates. ^∗^*P* < 0.05 based on ANOVA, followed by Tukey’s *post hoc* test **(B,C)** and two-tailed *t*-test **(E)**.

Because lncRNAs can modulate the expression of nearby PCGs (Batista and Chang, 2013; Uchida and Dimmeler, 2015), we tested the possibility that RAMP2-AS1 regulates RAMP2 expression in ECs. To inhibit RAMP2-AS1, we designed two locked nucleic acid (LNA) GapmeRs targeting two specific regions in Exons 2 and 4 of the Transcript 1, which are shared by Transcript 2 (Figure 3A). While the Exon 4-targeting LNA (LNA1) significantly reduced the RAMP2-AS1 RNA level, the other LNA (LNA2) did not result in consistent data (Supplementary Figure 5E). Therefore, we used LNA1, and hereafter simplified as LNA, to inhibit RAMP2-AS1 in the rest of the experiments. In ECs treated with vehicle control (DMSO) or ATV, LNA inhibition of RAMP2-AS1 led to a significant decrease in RAMP2 mRNA level (Figures 3B,C). The suppressive effect of RAMP2-AS1 was more profound and consistent in the DMSO-treated than ATV-treated ECs. Therefore, we focused on examining the effect of RAMP2-AS1 knockdown on RAMP2 and EC functions at baseline condition. In line with the changes at the RNA level, RAMP2-AS1 knockdown also caused a significant suppression in RAMP2 protein level (Figures 3D,E). These data suggest that RAMP2-AS1 regulates the expression of RAMP2 in ECs. Interestingly, RAMP-AS1 knockdown did not suppress the ratio of induction by ATV, for either RAMP2-AS1 or RAMP2 (Supplementary Figures 5F,G), suggesting the residual RAMP2-AS1 was still able to respond to ATV and RAMP2-AS1 suppression may activate other compensatory pathways contributing to ATV-induced RAMP2.

### Knock-Down of RAMP2-AS1 Increases EC Senescence and Decreases Angiogenic Capacity

**FIGURE 4:**
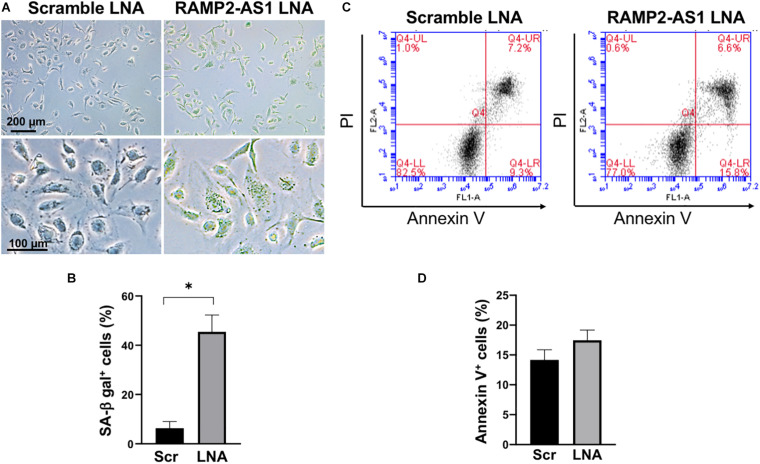
RAMP2-AS1 inhibition promotes cell senescence. HUVECs were transfected with scramble or RAMP2-AS1 LNA GapmeRs and then used for SA-β-gal staining and apoptosis assay. **(A)** Representative images of SA-β-gal staining (green indicates positive staining) of ECs transfected with scramble or RAMP2-AS1 LNA GapmeRs. Scale bars: 200 μm (upper images) and 100 μm (lower images). **(B)** Three randomly selected views were captured from each of the duplicates per condition per experiment and percentage of ECs with SA-β-gal positive staining were calculated. ^∗^*P* < 0.05 based on Student’s *t*-test. Data represent mean ± SEM from five independent experiments. **(C,D)** Apoptotic cells were quantified by Annexin V staining using flow cytometry. Representative scatter plots show in **(C)** and quantification of Annexin V^+^ cells expressed in percentage (%) in **(D)**. Data represent mean ± SEM based on three independent experiments.

**FIGURE 5:**
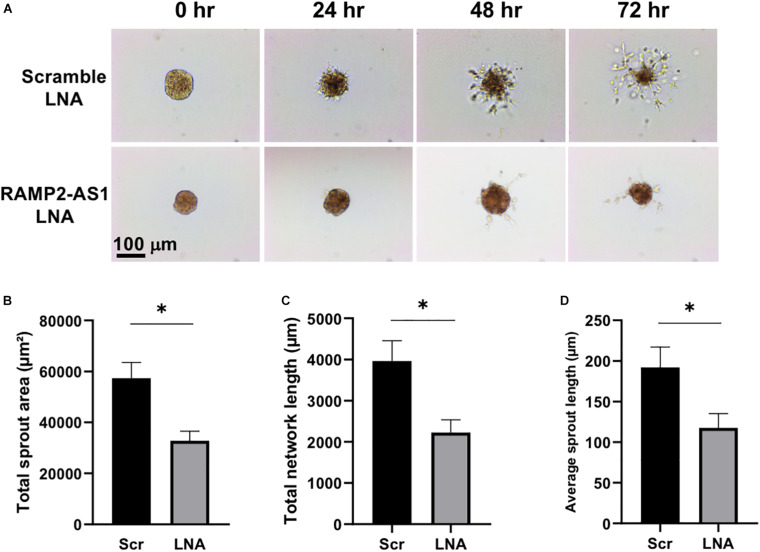
RAMP2-AS1 inhibition impairs EC angiogenic capacity. HUVECs were transfected with scramble or RAMP2-AS1 LNA GapmeRs for 48 h and then used in 3D spheroid sprouting assay. **(A)** Representative images showing the sprouting from the EC aggregates. **(B–D)** Quantitative analysis of EC sprouts after 72 h in hydrogel using three parameters as indicated. Ten aggregates were analyzed per condition in each experiment. *p < 0.05 based on Student’s *t*-test. Data are represented as mean ± SEM from three independent experiments.

Next, we investigated the effect of RAMP2-AS1 knockdown on EC functions, specifically cell senescence, apoptosis, and angiogenesis, all of which have been shown to be regulated by RAMP2 and related to vascular aging (Ichikawa-Shindo et al., 2008; Koyama et al., 2013). Senescence-associated β-galactosidase (SA-β-gal) staining, a marker of senescent cells, was increased in ECs with RAMP2-AS1 knockdown, as compared to ECs transfected with scramble control (Figures 4A,B). EC apoptosis, as assayed by Annexin V staining, was not significantly altered but showed a trend toward increase in ECs with RAMP2-AS1 knockdown (Figures 4C,D). When these cells were subjected to 3D spheroid sprouting assay, ECs transfected with scramble LNA began to sprout by 24 h and infiltrated into the surrounding hydrogel uniformly to form networks by 48 h. In contrast, ECs transfected with RAMP2-AS1 LNA show almost no sprouting by 24 h, very few stalks of cells extending from the central aggregate by 48 h, and no network formed by 72 h (Figure 5A). Quantitative analysis revealed that the total sprout area, total network length, and average sprout length were all significantly lower in the ECs with RAMP2-AS1 knockdown (Figures 5B–D). Taken together, these data support that RAMP2-AS1 is essential for EC homeostasis and the mechanisms involve the suppression of senescence and promotion of angiogenesis.

### Gene Network Regulated by RAMP2-AS1 in ECs

**FIGURE 6:**
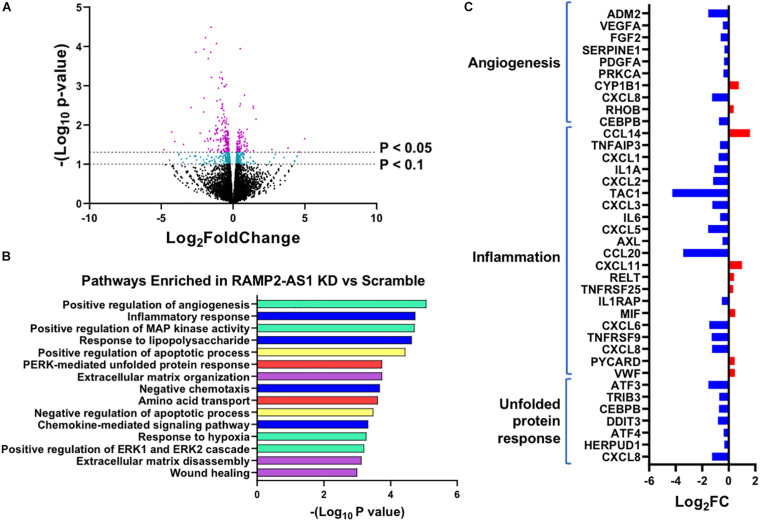
RNA-seq profiling of transcriptome change caused by RAMP2-AS1 knockdown. HUVECs were transfected with RAMP2-AS1 LNA or scramble control in biological replicates. **(A)** Number of genes showing DE in RNA-seq with indicated *P*-value cutoffs. Genes showing DE with *P* < 0.1 are in cyan and those with *P* < 0.05 are in purple. **(B)** Top 15 enriched pathways ranked by -log_10_ (*p*-value). Pathways belonging to the same family are grouped into similar colors; angiogenesis in cyan, inflammatory response in blue, unfolded protein response pathways in red, apoptosis in yellow, and ECM remodeling in purple. **(C)** Log2 FC (RAMP2-AS1 LNA vs. scramble control) of select genes involved in angiogenesis, inflammation, or unfolded protein response in RAMP2-AS1 knockdown vs. scramble control.

To gain additional information for RAMP2-AS1-regulated transcriptome, we performed RNA-seq in ECs transfected with scramble control or LNA inhibiting RAMP2-AS1. RNA-seq analysis identified 252 differential expressed genes (DEGs) with *P* < 0.05 and 585 DEGs with *P* < 0.1 in ECs with RAMP2-AS1 knockdown vs. scramble control, without a fold change cutoff (Figure 6A). To obtain an overview of cellular pathways affected by RAMP2-AS1 inhibition, we used the 585 DEGs with *P* < 0.1 for the subsequent pathway enrichment analysis. These included 232 upregulated and 353 downregulated genes. GO-based pathway enrichment analysis of these DEGs revealed their involvement in angiogenesis, inflammatory response, apoptosis, unfolded protein response, extracellular matrix (ECM) organization, and amino acid transportation (Figure 6B). In line with the impaired sprouting capacity (Figure 5), the inhibition of RAMP2-AS1 led to an overall downregulation of pro-angiogenic genes, including *VEGFA*, *PDGFA, FGF*, and *ADM2* (Figure 6C), supporting the effect observed with spheroid sprouting assay (Figure 5). In contrast, several genes encoding pro-inflammatory and coagulatory cytokines, e.g., *VWF*, *CXCL11*, *CCL14*, and *MIF*, were upregulated under RAMP2-AS1 knockdown, although some others are downregulated in the same condition. This lack of a clear direction in inflammation was in line with the insignificant effect of RAMP2-AS1 LNA in monocyte adhesion to ECs (data not shown). Interestingly, RAMP2-AS1 knockdown also caused a list of genes involved in unfolded protein response (UPR) to be downregulated (Figure 6C), which provides possible explanation to the increased EC senescence (Figure 4). Other genes differentially expressed due to RAMP2-AS1 inhibition are listed in Supplementary Table 2. Collectively, inhibition of RAMP2-AS1 affect multiple pathways essential for EC homeostasis.

## Discussion and Conclusion

In the current study, we employed PCA to identify crucial genes for divergent EC transcriptional states under PS vs. OS over time. Admittedly, EC aging encompasses many aspects of molecular and cellular changes, mediated through multiple levels of regulation other than transcriptional regulation. For example, DNA methylation has been used to calculate and predict epigenetic aging in several tissues and organs (Horvath and Raj, 2018). Furthermore, the relevance of using *in vitro* EC culture subjected to flow for up to 24 h to model the *in vivo* vascular aging that occurs in decades is another limitation. Nonetheless, the EC aging trajectories we have identified herein are an attempt to quantify EC transcriptional state over time in an unbiased manner by considering the genome-wide transcriptome changes, rather than a select set of genes.

PCA maximally preserves information and allows deconvolution of transcriptional profiles into interpretable components associated with the divergence in cells under various conditions. In particular, using PC3 of PCGs and PC2 of NCGs, we observed divergent trajectories of ECs under the PS vs. OS conditions. The divergence was supported by RNA-seq data from ATV and TNFα-treated EC, which, respectively, mapped close to PS and OS on PC3. Many of the genes identified from the PC3 of PCGs are well characterized as flow-responsive and essential for EC homeostasis, e.g., KLF2 and KLF4, the key transcription factors in ECs, and eNOS, the hallmark for EC homeostasis. Furthermore, many genes that have not been previously identified to be important regulators in flow-modulated EC function were also revealed by PC3, e.g., RAMP2. We also used mutual information-based statistics to analyze genes that may explain the flow-modulated distinct EC transcriptome profiles (Supplementary Table 3). Notably, many highly ranked genes derived from PCA, such as KLF2 and KLF4, are also highly ranked by mutual information (KLF ranked #1 and KLF2 ranked #12). Therefore, the PCA-identified PCGs and NCGs whose differential expressions under PS vs. OS contribute to the divergent aging trajectories are likely novel and important molecules regulating EC function.

Following a series of integrative bioinformatics analyses, we identified RAMP2-AS1 as a candidate novel lncRNA for further investigation. This is based on its top ranking in the degree of DE in ECs under various stimuli, as well as its highly correlated expression pattern with a key EC-regulator RAMP2 in cultured ECs and in human donor-derived arterial ECs. With respect to EC function, RAMP2-AS1 inhibition resulted in increased EC senescence and suppressed angiogenic sprouting function. At the molecular level, RAMP2-AS1 inhibition caused profound changes in a number of pathways, including angiogenesis, innate immune, and pro-inflammation activation, ECM organization, cell apoptosis, UPR, and amino acid transport. Of note, *ADM2* is also greatly downregulated by RAMP2-AS1 knockdown, suggesting that RAMP2-AS1 regulates ADM-RAMP2 signaling and EC homeostasis. On the other hand, we did not observe a significant effect in apoptosis and monocyte adhesion by RAMP2-AS1 knockdown. This is likely due to the baseline and static condition used in the present work. Future study is warranted to elucidate the role of RAMP2-AS1 in EC function in response to specific stimuli, such as different flow patterns and TNFα.

Given the genomic proximity of RAMP2-AS1 and RAMP2, and the nuclear localization of RAMP2-AS1 determined by the recently developed APEX-seq (Fazal et al., 2019), it is plausible that RAMP2-AS1 RNA acts *in cis* as a transcriptional activator for RAMP2, which is reminiscent of other lncRNAs (Kopp and Mendell, 2018). In support of this hypothesis, RAMP2-AS1 and RAMP2 are regulated in a highly correlational pattern. Moreover, RAMP2-AS1 knockdown strongly suppressed RAMP2 both at mRNA and protein levels. Importantly, EC functions previously identified to be regulated by RAMP2, e.g., angiogenesis and senescence (Ichikawa-Shindo et al., 2008; Koyama et al., 2013), were affected in a similar fashion by the RAMP2-AS1 knockdown. However, it is also possible that RAMP2-AS1 can function *in trans* to affect the transcription of other genes, as demonstrated for other nuclear-localized lncRNAs in ECs (Miao et al., 2018; Calandrelli et al., 2020). The molecular mechanisms involving RAMP2-AS1 with expanded scope of EC biology are of interest for future studies.

In conclusion, herein we exploited PCA to dissect the temporal and transcriptomic changes underlying the distinct effects elicited by physiological *vs.* pathophysiological flow in ECs. Integrative analysis of the divergent aging trajectories of ECs subject to PS *vs.* OS, TNFα, and ATV revealed RAMP2 and RAMP2-AS1 as novel regulators in EC aging and function. Suppression of RAMP2-AS1 leads to decreased RAMP2 expression, impaired EC sprouting, and increased EC senescence. Our study presents a novel systems biology approach to identify potential regulators in EC aging, as exemplified by RAMP2-AS1.

## Data Availability Statement

The datasets presented in this study can be found in online repositories. The names of the repository/repositories and accession number(s) can be found below: https://www.ncbi.nlm.nih.gov/geo/, Accession IDs: GSE103672, GSE136912, and GSE163433.

## Ethics Statement

The research consents for the use of postmortem human tissues were obtained from the donors’ next of kin and ethical approval for this study was granted by the Institutional Review Board of City of Hope (IRB #01046).

## Author Contributions

C-HL, RR, and ZC conceived the research. C-HL, AC, AB, RR, KS, YL, XT, SB, S-LC, and DO’M designed and performed experiments and analyzed the data. ZC, RR, and P-HH supervised experiments and interpreted results. C-HL, AC, AB, KS, RR, and ZC wrote the manuscript. SC and JS revised the manuscript. ZC and RR obtained funding for this study. All authors contributed to the article and approved the submitted version.

## Conflict of Interest

The authors declare that the research was conducted in the absence of any commercial or financial relationships that could be construed as a potential conflict of interest.
