# Supplementary material for: RAMP2-AS1 Regulates Endothelial Homeostasis and Aging
Source: Front Cell Dev Biol. 2021 Feb 12;9:635307. doi: 10.3389/fcell.2021.635307 (PMC7907448; doi:10.3389/fcell.2021.635307)
Supplement: Supplementary file 1 [file Data_Sheet_1.docx]

**Supplementary Material**

**Supplementary Figure 1**

**Principal Component Analysis of Time-Series RNA-seq data.**  (**A)** All principal components of the data matrix composed of time-series RNA-Sequencing data for protein coding genes collected from OS and PS conditions. An elbow is identified at component 8, indicating a majority of the variance and information are contained in the first 8 components. (**B)** The first 8 principal components plotted against each other. Data in OS condition is shown in black; PS in red. The diagonal shows a grouped histogram of the data along the column. Principal component 4 plotted against PC3 (blue box) shows the best separation between OS and PS conditions. PC3 was used to identify divergent aging trajectories between the two conditions. (**C)** The first 8 principal components plotted over time in OS and PS conditions. The PS and OS time trajectories diverge in PC4 for protein coding genes. PC1 is correlated with time, PC2 shows intermittent spiking which is associated with mitochondrial gene expression, PC3 shows divergent trajectories between PS and OS conditions. The other components are not clearly interpretable. (**D)** Same as is shown in (**C)** for non-coding genes. The divergent trajectories associated with aging are shown in PC2.

**Supplementary Figure 2**

**EC aging trajectories under ATV and Hypoxia conditions.** RNA-seq was performed on HUVECs treated with ATV at 5 μM for 24 hr or TNFα **(A)** and human microvascular ECs subject to 2% O2 (hypoxia) for different time points as indicated **(B)**. The third principal component loading vector given by the third column of the matrix *V** in the singular value decomposition $X=U\Sigma V^{*}$ corresponding to PC3 in the protein coding genes are used to map data from **(A)** ATV and TNFα and **(B)** hypoxic conditions into the PS vs. OS aging trajectory space.

**Supplementary Figure 3**

**The workflow for identifying candidate lncRNAs.**

**Supplementary Figure 4**


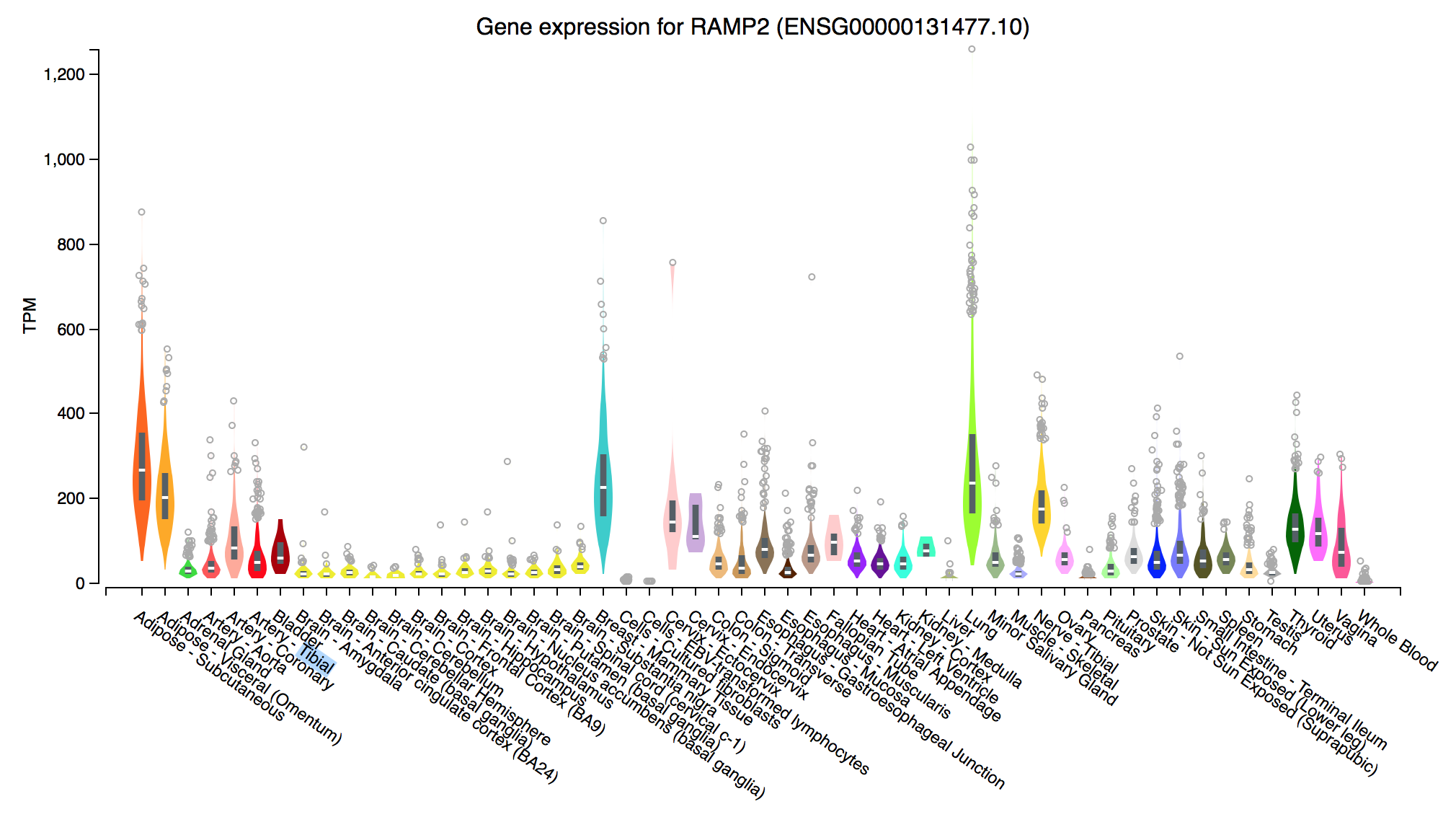

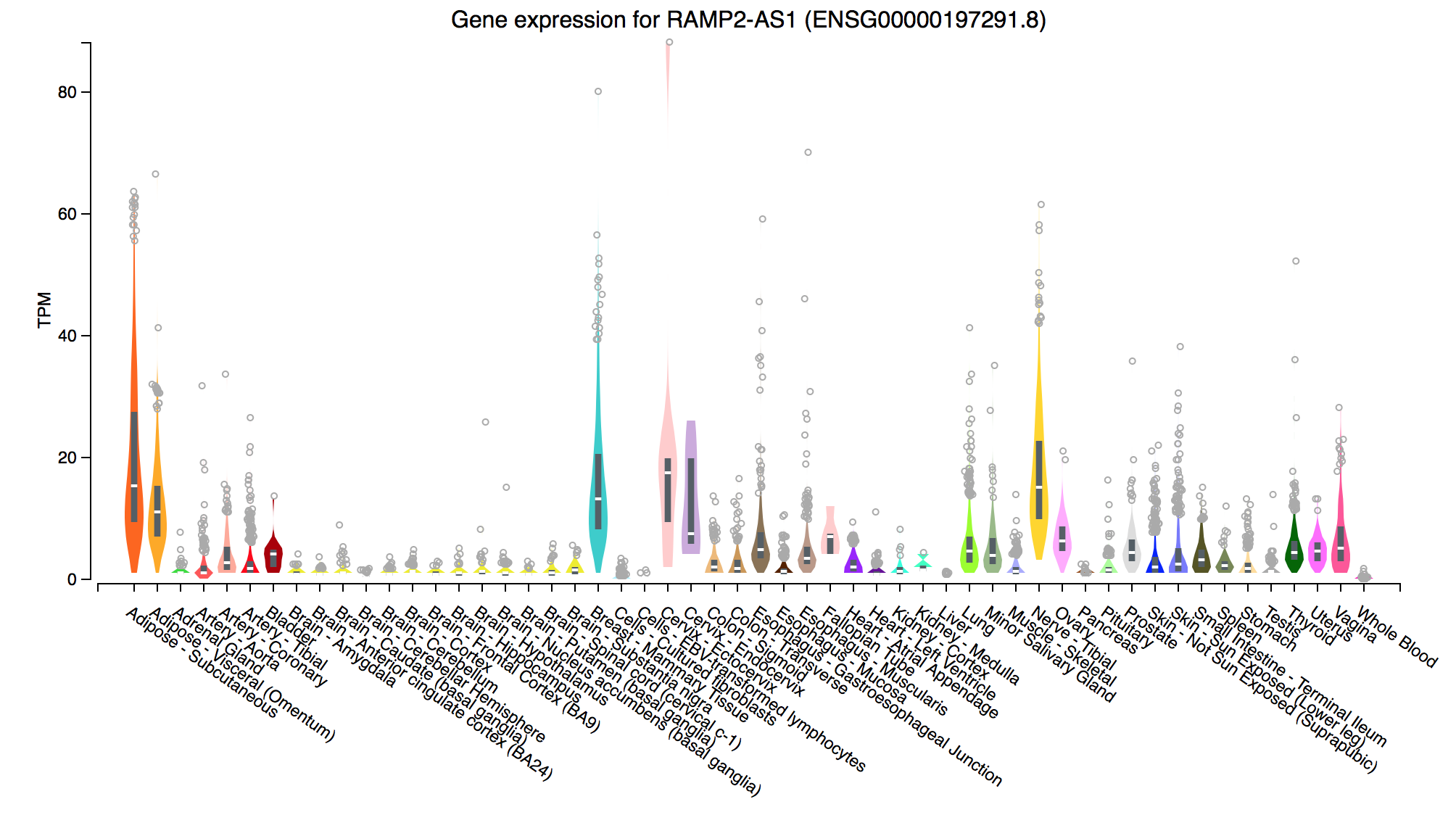


**Gene expression of RAMP2 and RAMP2-AS1 in various tissues based on GTEX.**

**Supplemental Figure 5**

**RAMP2-AS1 primer and LNA design and testing.** **(A)** Primer design for RAMP2-AS1. **(B)** Primer testing using 4 sets of primers in HUVEC treated with indicated conditions. **(C,D)** qPCR analysis of RAMP2-AS1 (in C) and RAMP2 (in D) RNA levels in HUVECs without treatment (NTC) or treated with TNFα at 10, 20, or 100 ng/mL for 24 hours. The respective RNA level in NTC was set as 1. **(E)** HUVECs were transfected with respective LNAs targeting RAMP2-AS1. The level of RAMP2-AS1 was detected by qPCR, with ACTB as internal control. **(C-E)** *P < 0.05 between indicated groups. Data represent mean ± SEM from three independent experiments. (**F,G**) Bar graphs showing the comparison of the ratio of ATV/DMSO in scramble group to that in LNA group for RAMP2-AS1 (in F) and RAMP2 (in G), based on data shown in Fig. 3, B and C.

**Supplementary Figure 6**

**Donor characteristics for intimal RNA quantification of RAMP2 and RAMP2-AS1.** Among 44 donors in total, the age is 41± 2 year-old **(A)** and BMI is 29.8 ± 0.9 **(B)**. There were 32 male and 12 female donors **(C)**.

**Supplementary Table 1**. PCA-identified top 50 PCGs and NCGs contributing to divergent PS vs OS trajectories. RAMP2 and RAMP2-AS1 are boldfaced.

| **Rank** | **PCG** | **NCG** |
| --- | --- | --- |
| 1 | CD34 | MIR503HG |
| 2 | ITGB4 | C1orf132 |
| 3 | LCNL1 | LINC01358 |
| 4 | PTGDS | RP1-117P20.3 |
| 5 | RP11-229P13.28 | RP11-26J3.1 |
| 6 | CYP1B1 | RP11-201A3.1 |
| 7 | SLCO2A1 | LINC01629 |
| 8 | KLF4 | AC122136.2 |
| 9 | SOX18 | RP11-631N16.2 |
| 10 | ASS1 | RP11-2B6.2 |
| 11 | APOL3 | **RAMP2-AS1** |
| 12 | NPR1 | MIR137HG |
| 13 | KLF2 | CTC-339F2.2 |
| 14 | ABCG2 | RP11-534C12.1 |
| 15 | KAZALD1 | RP11-696D21.2 |
| 16 | CXCR4 | RP11-672A2.4 |
| 17 | HR | RP11-1100L3.8 |
| 18 | **RAMP2** | RP11-131L23.2 |
| 19 | SULF1 | NEAT1 |
| 20 | SLC9A3R2 | LINC00520/LEENE |
| 21 | CLDN5 | RP11-356J5.12 |
| 22 | FCN3 | RP11-113K21.4 |
| 23 | HOXD1 | RP11-253E3.3 |
| 24 | PI16 | RP11-433J22.3 |
| 25 | APLN | WI2-87327B8.2 |
| 26 | PCDH19 | RP11-66B24.1 |
| 27 | CYP1A1 | RP11-66B24.2 |
| 28 | HAGHL | RP11-12G12.7 |
| 29 | DKK2 | RP11-254F7.2 |
| 30 | ZNF467 | EPB41L4A-AS1 |
| 31 | FGFR3 | CTC-246B18.10 |
| 32 | DHH | RP11-54A9.1 |
| 33 | CNR1 | RP11-775C24.5 |
| 34 | PCDH12 | RP5-1086K13.1 |
| 35 | ACKR3 | LINC01235 |
| 36 | TSPAN18 | PVT1 |
| 37 | VIPR1 | RP11-879F14.1 |
| 38 | ACP5 | RP11-879F14.2 |
| 39 | CRACR2B | RP11-879F14.3 |
| 40 | TGM2 | RP11-923I11.3 |
| 41 | MMP10 | RP11-51J9.5 |
| 42 | TNFSF10 | CTD-2134A5.3 |
| 43 | HEG1 | LINC01013 |
| 44 | PTGIS | LINC00467 |
| 45 | LIMS2 | LINC00632 |
| 46 | ST8SIA6 | RP11-147L13.11 |
| 47 | SIGIRR | RP11-147L13.12 |
| 48 | ATG9B | RP11-147L13.13 |
| 49 | THBD | RP11-358B23.1 |
| 50 | ALPL | RP11-334C17.6 |

**Supplementary Table 2. RNA-seq identified 252 DEGs in HUVECs with RAMP2-AS1 knockdown, ranked by log2 FC.**

| **Gene** | **Base Mean reads** | **log2FC** |
| --- | --- | --- |
| CCDC73 | 3.08 | 4.99 |
| BX255925.4 | 2.37 | 4.61 |
| SPATA21 | 4.07 | 3.83 |
| AC022966.3 | 6.02 | 2.71 |
| CCL14 | 36.08 | 1.60 |
| AL121748.1 | 17.53 | 1.43 |
| RN7SL1 | 114.00 | 1.42 |
| WTAPP1 | 45.05 | 1.40 |
| TENM4 | 34.41 | 1.05 |
| CXCL11 | 49.87 | 1.00 |
| ABCA9 | 161.71 | 0.98 |
| ITGA11 | 146.61 | 0.98 |
| CYP26B1 | 137.71 | 0.97 |
| AC011603.3 | 57.45 | 0.93 |
| AC022075.1 | 116.38 | 0.86 |
| MMP11 | 164.11 | 0.86 |
| AL158206.1 | 64.23 | 0.83 |
| LUM | 96.03 | 0.81 |
| MTMR9LP | 124.50 | 0.80 |
| CYP1A1 | 323.91 | 0.80 |
| AL133415.1 | 130.39 | 0.78 |
| VIPR1 | 76.07 | 0.76 |
| GDPD5 | 364.67 | 0.73 |
| CHST7 | 278.38 | 0.71 |
| CASTOR3 | 170.99 | 0.63 |
| ALDH1A1 | 1565.47 | 0.60 |
| F2RL3 | 243.98 | 0.59 |
| ANO8 | 269.79 | 0.58 |
| CLDN5 | 1461.32 | 0.58 |
| PIM3 | 4125.43 | 0.58 |
| GSTO2 | 146.26 | 0.56 |
| TSPAN13 | 710.40 | 0.56 |
| SMAD6 | 1074.74 | 0.52 |
| TMSB4X | 6640.14 | 0.50 |
| ADAMTS7 | 2348.48 | 0.50 |
| MSMP | 399.13 | 0.50 |
| LMO2 | 557.99 | 0.49 |
| C4orf48 | 241.28 | 0.48 |
| TXNDC5 | 857.14 | 0.48 |
| FAM89B | 269.38 | 0.48 |
| LINC00607 | 685.54 | 0.48 |
| VWF | 47107.27 | 0.48 |
| FAM124B | 1944.44 | 0.47 |
| CDC42EP5 | 223.00 | 0.47 |
| MYORG | 203.36 | 0.47 |
| SEMA4C | 516.04 | 0.46 |
| PPM1K | 320.76 | 0.46 |
| SLC40A1 | 833.49 | 0.46 |
| LIMD2 | 782.05 | 0.45 |
| KCTD12 | 15017.82 | 0.45 |
| SH3TC1 | 2704.52 | 0.45 |
| ARSA | 459.96 | 0.45 |
| ADAMTS4 | 1804.60 | 0.44 |
| SPPL2B | 391.88 | 0.44 |
| PGGHG | 397.14 | 0.42 |
| GPR137 | 391.28 | 0.42 |
| TMEM179B | 417.08 | 0.42 |
| FAM102A | 769.02 | 0.41 |
| SUOX | 433.80 | 0.41 |
| RELT | 449.46 | 0.40 |
| TP53I11 | 2845.36 | 0.40 |
| DIPK1B | 1949.23 | 0.40 |
| RPS2P5 | 681.03 | 0.39 |
| HTRA1 | 4169.65 | 0.38 |
| MEGF6 | 1411.45 | 0.38 |
| SPNS2 | 811.95 | 0.38 |
| SELENOW | 2737.23 | 0.36 |
| DNAJB4 | 1725.62 | 0.36 |
| NAGLU | 696.48 | 0.35 |
| TCIRG1 | 1166.67 | 0.34 |
| PCDH10 | 6100.48 | 0.33 |
| MAN2C1 | 1189.12 | 0.31 |
| VPS51 | 1159.27 | 0.31 |
| COPE | 1277.47 | 0.30 |
| MGAT1 | 2609.28 | 0.30 |
| LTBP3 | 2825.11 | 0.29 |
| HYAL2 | 5319.24 | 0.29 |
| MCRIP1 | 962.98 | 0.29 |
| MEG3 | 4313.32 | 0.28 |
| ID3 | 2624.67 | 0.28 |
| MTATP6P1 | 3007.21 | 0.27 |
| TSPO | 2251.82 | 0.27 |
| NBEAL2 | 3469.49 | 0.26 |
| IGF2BP2 | 2363.68 | -0.26 |
| ASAP1 | 5547.69 | -0.29 |
| TARS1 | 5072.95 | -0.29 |
| EIF5 | 7867.81 | -0.30 |
| TRIB1 | 794.88 | -0.30 |
| SLFN5 | 3134.25 | -0.30 |
| MARS1 | 3894.15 | -0.30 |
| SARS1 | 3861.58 | -0.31 |
| SLC38A1 | 11173.84 | -0.32 |
| TRIM56 | 1525.07 | -0.33 |
| SERPINE1 | 130234.62 | -0.33 |
| FLOT1 | 2160.04 | -0.33 |
| CITED2 | 1585.54 | -0.33 |
| TAOK3 | 1414.85 | -0.34 |
| MTHFD1L | 2913.58 | -0.34 |
| XPOT | 4456.48 | -0.34 |
| VRK2 | 1071.66 | -0.34 |
| CDC42EP1 | 2834.23 | -0.34 |
| HERPUD1 | 1136.10 | -0.34 |
| AARS1 | 3244.86 | -0.34 |
| CCN2 | 22266.54 | -0.34 |
| AC022966.1 | 1720.04 | -0.34 |
| GLIPR1 | 1729.00 | -0.35 |
| IARS1 | 7226.57 | -0.35 |
| MTND4P12 | 992.01 | -0.36 |
| PDGFA | 565.44 | -0.36 |
| YARS1 | 3217.58 | -0.36 |
| CDYL | 969.83 | -0.36 |
| RPF2 | 636.29 | -0.37 |
| MAP1LC3B | 4315.58 | -0.37 |
| SAMD4A | 2884.95 | -0.37 |
| ESM1 | 5484.93 | -0.37 |
| MAP7D3 | 932.55 | -0.37 |
| GPR4 | 1918.75 | -0.38 |
| SLC7A11 | 10615.97 | -0.38 |
| N4BP2 | 771.58 | -0.38 |
| NOTCH2 | 4677.36 | -0.38 |
| NMT2 | 2109.76 | -0.38 |
| LYAR | 792.47 | -0.39 |
| SQSTM1 | 14172.74 | -0.39 |
| EDN1 | 4169.66 | -0.39 |
| ATF4 | 3119.12 | -0.39 |
| EBNA1BP2 | 2346.97 | -0.39 |
| RND3 | 2794.58 | -0.40 |
| TNFSF18 | 425.05 | -0.41 |
| SERPINE2 | 728.94 | -0.41 |
| TM6SF1 | 721.11 | -0.41 |
| FILIP1L | 634.26 | -0.41 |
| CD44 | 12423.50 | -0.41 |
| TENM3 | 2525.67 | -0.41 |
| NAV3 | 2808.20 | -0.42 |
| PSPH | 433.50 | -0.42 |
| HK2 | 806.90 | -0.42 |
| ERRFI1 | 1019.21 | -0.42 |
| IFRD1 | 979.58 | -0.43 |
| NCR3LG1 | 779.64 | -0.44 |
| TFEC | 1405.50 | -0.44 |
| VEGFA | 683.18 | -0.45 |
| SYNE1 | 2061.58 | -0.45 |
| PLOD2 | 13566.10 | -0.45 |
| ABITRAM | 299.72 | -0.46 |
| LACC1 | 240.47 | -0.47 |
| DNAJC1 | 637.27 | -0.47 |
| SPRY2 | 767.69 | -0.47 |
| MXD1 | 495.67 | -0.47 |
| GARS1 | 7413.53 | -0.47 |
| LINC02693 | 996.79 | -0.47 |
| AXL | 8363.91 | -0.48 |
| MT1E | 641.58 | -0.48 |
| LNCOG | 338.68 | -0.48 |
| SLC1A5 | 3534.36 | -0.49 |
| NEXN | 654.41 | -0.50 |
| TXNRD1 | 12851.40 | -0.51 |
| E2F7 | 2215.97 | -0.51 |
| BNC1 | 970.89 | -0.52 |
| LAMB3 | 501.91 | -0.53 |
| NR3C1 | 1729.73 | -0.53 |
| PCK2 | 500.56 | -0.54 |
| DCBLD2 | 8508.03 | -0.54 |
| FAM210A | 483.61 | -0.54 |
| PSAT1 | 2484.15 | -0.55 |
| NFIL3 | 384.58 | -0.55 |
| SLIT2 | 1713.54 | -0.55 |
| ADTRP | 1313.96 | -0.56 |
| CLIP4 | 726.64 | -0.57 |
| TMEM158 | 591.32 | -0.57 |
| RUNX1 | 2147.28 | -0.57 |
| NR3C2 | 709.53 | -0.57 |
| ADAM12 | 167.14 | -0.58 |
| PDCD1LG2 | 302.92 | -0.58 |
| CREB5 | 249.18 | -0.60 |
| AJUBA | 1438.10 | -0.60 |
| STC2 | 3370.02 | -0.61 |
| FGF2 | 245.02 | -0.62 |
| MTHFD2 | 2582.94 | -0.62 |
| ANKRD1 | 11297.49 | -0.64 |
| SGK1 | 4674.14 | -0.65 |
| GEM | 221.22 | -0.65 |
| IL6 | 170.28 | -0.66 |
| TNFAIP3 | 326.73 | -0.66 |
| DDIT4L | 256.25 | -0.68 |
| CD274 | 436.83 | -0.69 |
| PDGFC | 533.92 | -0.70 |
| CCNA1 | 357.26 | -0.72 |
| TRIB3 | 1869.23 | -0.73 |
| ANKRD18B | 320.81 | -0.73 |
| KLF11 | 242.99 | -0.74 |
| CEBPB | 454.03 | -0.74 |
| MIR100HG | 963.60 | -0.75 |
| RASGRF2 | 1129.47 | -0.75 |
| DDIT4 | 1261.32 | -0.75 |
| CXCL1 | 500.44 | -0.77 |
| LURAP1L | 177.79 | -0.77 |
| GADD45B | 326.09 | -0.78 |
| KCTD16 | 197.03 | -0.80 |
| DDIT3 | 310.80 | -0.82 |
| SLC7A5 | 2223.37 | -0.82 |
| G0S2 | 165.83 | -0.84 |
| NRXN3 | 312.11 | -0.84 |
| LAMP3 | 126.40 | -0.84 |
| SLC8A1 | 161.22 | -0.88 |
| PAPPA2 | 156.16 | -0.88 |
| P3H2 | 110.90 | -0.89 |
| ALDH1L2 | 144.34 | -0.90 |
| SNAPC1 | 629.70 | -0.93 |
| IL7R | 215.84 | -0.96 |
| CCDC122 | 46.09 | -1.00 |
| CECR2 | 40.65 | -1.01 |
| CHAC1 | 268.61 | -1.01 |
| ELFN2 | 75.26 | -1.02 |
| CCDC190 | 143.31 | -1.06 |
| LINC00973 | 101.34 | -1.07 |
| NMRAL2P | 62.33 | -1.09 |
| RAB3IL1 | 66.68 | -1.09 |
| IL1A | 87.03 | -1.10 |
| AFF3 | 183.14 | -1.12 |
| ULBP1 | 77.88 | -1.13 |
| IRS1 | 78.09 | -1.14 |
| NIBAN1 | 589.80 | -1.15 |
| FENDRR | 87.02 | -1.15 |
| CXCL2 | 77.32 | -1.18 |
| IL20RB | 62.44 | -1.20 |
| CXCL3 | 37.52 | -1.24 |
| SPX | 50.71 | -1.26 |
| CCBE1 | 26.86 | -1.35 |
| SEMA3C | 33.62 | -1.45 |
| SLC6A9 | 117.17 | -1.50 |
| ATF3 | 322.07 | -1.54 |
| ADM2 | 165.42 | -1.55 |
| CXCL5 | 30.88 | -1.56 |
| PIANP | 15.04 | -1.57 |
| H2BC8 | 19.77 | -1.58 |
| FERMT1 | 20.40 | -1.64 |
| AC004771.2 | 13.10 | -1.70 |
| TRAF1 | 108.46 | -1.72 |
| RRAD | 19.49 | -1.74 |
| MSC | 19.92 | -1.77 |
| HIF1A-AS3 | 88.97 | -1.91 |
| AC089983.1 | 12.78 | -1.96 |
| MC5R | 28.57 | -2.01 |
| KIF1A | 75.16 | -2.01 |
| AL138828.1 | 24.22 | -2.04 |
| BIRC3 | 220.62 | -2.28 |
| CLDN1 | 44.93 | -2.59 |
| RPLP0P2 | 14.17 | -2.91 |
| CCL20 | 5.66 | -3.44 |
| UBE2MP1 | 4.14 | -4.05 |
| TAC1 | 4.78 | -4.26 |
| UBA52P5 | 2.43 | -4.80 |

**Supplementary Table 3.** Top 50 PCGs and NCGs contributing to divergent PS vs OS trajectories based on mutual information.

| **Rank** | **Gene** |
| --- | --- |
| 1 | KLF4 |
| 2 | EDN1 |
| 3 | LIMS2 |
| 4 | CNR1 |
| 5 | STARD8 |
| 6 | CXCR4 |
| 7 | SLC9A3R2 |
| 8 | NPR1 |
| 9 | ITGB4 |
| 10 | DHH |
| 11 | FGFR3 |
| 12 | KLF2 |
| 13 | C14orf80 |
| 14 | AL928654.7 |
| 15 | CRIP1 |
| 16 | RP5-900K19.2 |
| 17 | ARPC1B |
| 18 | ARPC1A |
| 19 | C3orf36 |
| 20 | ARHGEF15 |
| 21 | FAM65A |
| 22 | SLCO2A1 |
| 23 | TRNP1 |
| 24 | PPP1R3C |
| 25 | SYNPO |
| 26 | RNPEPL1 |
| 27 | GJC2 |
| 28 | NEXN |
| 29 | TNFRSF25 |
| 30 | PLEKHG5 |
| 31 | TNFSF15 |
| 32 | KIAA1522 |
| 33 | GNAZ |
| 34 | GIT1 |
| 35 | STMN3 |
| 36 | GNGT2 |
| 37 | TINAGL1 |
| 38 | TUSC2 |
| 39 | HYAL2 |
| 40 | TRIOBP |
| 41 | RP1-37E16.12 |
| 42 | NOL12 |
| 43 | ACVRL1 |
| 44 | DDIT4 |
| 45 | DRAXIN |
| 46 | CHST3 |
| 47 | PIM3 |
| 48 | SIGIRR |
| 49 | DNASE1L1 |
| 50 | FGF2 |
